# Efficiency of an Active Rehabilitation Intervention in a Slow-to-Recover Paediatric Population following Mild Traumatic Brain Injury: A Pilot Study

**DOI:** 10.1155/2016/5127374

**Published:** 2016-12-18

**Authors:** Sarah Imhoff, Philippe Fait, Frédérike Carrier-Toutant, Geneviève Boulard

**Affiliations:** ^1^Department of Human Kinetics, Université du Québec à Trois-Rivières (UQTR), 3351 Boulevard des Forges, Trois-Rivières, QC, Canada G9A 5H7; ^2^Research Group on Neuromusculoskeletal Dysfunctions (GRAN), UQTR, 3351 Boulevard des Forges, Trois-Rivières, QC, Canada G9A 5H7; ^3^Cortex Médecine et Réadaptation Concussion Clinic, 205-1035 Avenue Wilfrid-Pelletier, Québec City, QC, Canada G1W 0C5; ^4^Research Center in Neuropsychology and Cognition (CERNEC), Université de Montréal, 90 rue Vincent d'Indy, Montréal, QC, Canada H2V 2S9; ^5^Clinique Neuropsychologique, 206-1379 Chemin Ste-Foy, Québec City, QC, Canada G1S 2N2

## Abstract

*Objective*. The aim of this study was to identify whether the addition of an individualised Active Rehabilitation Intervention to standard care influences recovery of young patients who are slow-to-recover following a mTBI.* Methods*. Fifteen participants aged 15 ± 2 years received standard care and an individualised Active Rehabilitation Intervention which included (1) low- to high-intensity aerobic training; (2) sport-specific coordination exercises; and (3) therapeutic balance exercises. The following criteria were used to measure the resolution of signs and symptoms of mTBI: (1) absence of postconcussion symptoms for more than 7 consecutive days; (2) cognitive function corresponding to normative data; and (3) absence of deficits in coordination and balance.* Results*. The Active Rehabilitation Intervention lasted 49 ± 17 days. The duration of the intervention was correlated with self-reported participation ($\overset{-}{x}=84.64\pm 19.63$%, *r* = −0.792, *p* < 0.001). The average postconcussion symptom inventory (PCSI) score went from a total of 36.85 ± 23.21 points to 4.31 ± 5.04 points after the intervention (*Z* = −3.18, *p* = 0.001).* Conclusion*. A progressive submaximal Active Rehabilitation Intervention may represent an important asset in the recovery of young patients who are slow-to-recover following a mTBI.

## 1. Introduction

Mild traumatic brain injury (mTBI), or concussion, is an acute neurological disorder induced after a direct or indirect mechanical impact to the head (acceleration, deceleration, rotational force, etc.) [1]. MTBI is the most common type of brain injury, and it represents a major health problem in children and adolescents, as they are highly represented in sport-related head injury [2–5]. The annual incidence of mTBI in Canadians is estimated at 600 per 100,000 inhabitants [6, 7]. Although recovery following mTBI is usually a short-lived process (7–10 days), physical, cognitive, and emotional symptoms and sleep disorder may persist beyond the expected recovery period of one to three months in 10–30% of mTBI cases in youth [8–11]. Postconcussion syndrome (PCS) refers to persisting symptoms for more than 1 month following a mTBI, including headache, nausea, dizziness, fatigue, vision impairment, sensitivity to noise and/or light, balance impairment, difficulty concentrating, memory impairment, drowsiness, anxiety, irritability, and feeling “in a fog” (WHO ICD-10). Barlow et al. [8] found that 58.5% of children aged from 0 to 18 years who sustained a mTBI were still symptomatic 1 month following the incident. Whereas children and adolescents presenting postconcussion symptoms beyond 1 month following a mTBI are considered slow-to-recover, there is a need to develop feasible and valid interventions to improve recovery in the paediatric population who sustained a mTBI.

Little is known about the treatment of mTBI, especially when postconcussion symptoms persist beyond the normal expected period of convalescence. Up to now, rest has been considered as “the gold standard” after sustaining a mTBI. Thus, consensus statements either recommend that athletes should rest until they are asymptomatic before introducing physical activity [12–14] or do not state on how and when physical activity should be introduced when symptoms persist beyond the expected recovery period [1, 15]. It has been suggested that aerobic exercise could restore cerebrovascular function and autonomic balance following mTBI [16–24]. Recent reviews of the literature support the benefits of exercise as an active rehabilitation treatment when postconcussion symptoms persist beyond the expected recovery period after injury [20, 21, 25, 26]. Indeed, it has been postulated that the introduction of physical activity may have a positive impact on the chronic symptoms following mTBI [17, 24, 27–42]. Preliminary evidence demonstrated that exercise, when used as a treatment, reduces reported symptoms in adults [17, 18, 22, 43, 44] and in youth who are slow-to-recover after mTBI [9, 35, 45]. For the sake of improving knowledge about the care of youth with atypical recovery following a mTBI, we studied the effectiveness of the integration of an Active Rehabilitation Intervention coupled with standard care while participants were still experiencing postconcussion symptoms at rest. Considering the lack of available scientific literature on active rehabilitation, we adapted the paediatric active rehabilitation program described by Gagnon et al. [9]. Our Active Rehabilitation Intervention differed in training frequency (3 times a week versus daily) and intensity reached by the end of the treatment.

The aim of this study was to identify whether the addition of an individualised Active Rehabilitation Intervention to standard care influences recovery of young patients who are slow-to-recover following an mTBI and still symptomatic at rest.

## 2. Methods

### 2.1. Participants

Eighteen participants aged from 10 to 17 years who complained of postconcussion symptoms for more than 4 weeks following a mTBI were referred to the Active Rehabilitation Intervention study through the* Cortex Medicine and Rehabilitation Concussion Clinic (Québec city, Qc, Canada)*. Participants received an interdisciplinary intervention consisting of rest, general education, school adaptations, and putting a halt to participation in physical activities, as proposed by the Zurich Consensus [1]. The study was explained to the families by a registered neuropsychologist during an initial 1-hour meeting in which the interdisciplinary approach was presented and the patient interview was conducted. Written parental consent and participant assent were obtained if both indicated an interest in the study. The research project was approved by the Ethics Review Board of Université du Québec à Trois-Rivières (UQTR).

### 2.2. Study Design

Participants underwent a full assessment looking for symptoms and neurological, balance, and coordination impairments before and after the Active Rehabilitation Intervention. The initial evaluation was conducted 49 ± 17 days after mTBI. A standardized mTBI history questionnaire was administered by a trained neuropsychologist to obtain detailed information about the mTBI history, including the date of the injury, the mechanism of injury, the clinical symptoms (loss of consciousness, confusion, and amnesia), and the number of autoreported previous mTBI. Participation in the program and self-report symptoms were assessed during a weekly follow-up phone call. Final evaluation was conducted when the participants reported being asymptomatic for a minimum of seven days. The following criteria were used to determine the resolution of signs and symptoms of mTBI: (1) absence of postconcussive symptoms for more than seven consecutive days; (2) cognitive function corresponding to normative data when assessed by a neuropsychologist; and (3) absence of deficits in coordination and balance.

### 2.3. Active Rehabilitation Intervention

**Figure 1:**
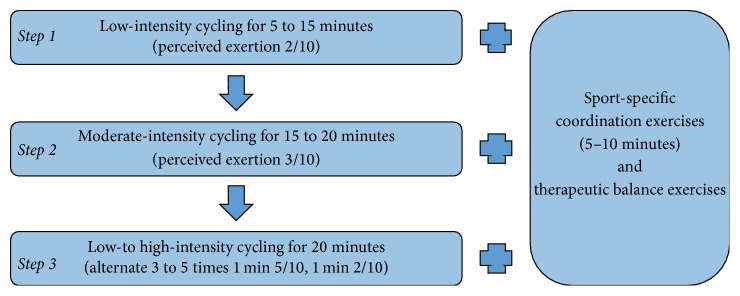
Active Rehabilitation Intervention.

Since participants were still presenting postconcussion symptoms at rest, they could not perform the standard 6-step gradual return to physical activities as proposed by the 2013 Zurich Consensus [1]. They were asked to perform a personalized home-based active rehabilitation program which consisted of (1) progressive submaximal low- to high-intensity (based on perceived exertion) aerobic training for up to 20 min, (2) low-intensity sport-specific coordination exercises for up to 10 min, and (3) therapeutic balance exercises (Figure 1). The active rehabilitation program was initially performed in a clinic under the supervision of a licensed kinesiologist. Participants were asked to perform the active rehabilitation program 3 times a week at home, at school, at a sport facility, or at the clinic. The individualised home-based program was inspired from the Montréal Children's Hospital Trauma Centre Concussion Clinic/Mild Traumatic Brain Injury Program (MCH) [9, 45], but differed in the frequency of participation (3 times a week in the present study versus daily at the MCH) in order to facilitate treatment compliance and minimize the impact on participants' daily life. This also enabled a greater difference in daily participation in exercises, allowing better monitoring of symptoms that may have been created through exercise since symptoms can be compared between a day without physical activity and the day where the rehabilitation program was conducted. It allowed assurances that the intervention was not harmful to the participant. This training schedule also enables a day of rest between high-intensity training periods. This allowed the exercise session to be more difficult without risking overtiredness for the participant and creating an increase in symptoms. Our Active Rehabilitation Intervention also differed in the intensity and duration of aerobic exercise reached by the end of the program, which was similar to those of rehabilitation programs conducted in adults. The intensity reached by the end of the program was therefore closer to the one usually performed in organized sports. This may promote a better transition to the sport practised by the participant and could enable a more complete rehabilitation. Patients were requested to limit the duration and intensity of exercises to those prescribed according to their clinical evaluation. They were asked to perform their program even if they were symptomatic at rest but had to stop the exercise if any of the current symptoms increased by 1 point on a symptom intensity scale from 0 to 10 points or at any onset of a new symptom. Participants had to regress one stage in the program if symptoms were exacerbated after exercise participation.

#### 2.3.1. Aerobic Training

Participants were asked to use an upright cycling ergometer to perform individualised progressive aerobic training program. Because of difficult access to a cycling ergometer, participant #7 used an elliptic. The level of perceived exertion was monitored using the 10-point Borg category-ratio scale (CR10), where effort is rated from none (0) to maximal (10) [46]. This scale was incorporated into the home program in order to help participants monitor exercise intensity. First, participants were asked to perform a symptom-dependent low-intensity supervised aerobic training session aiming to determine the volume at which they would initiate the aerobic training progression (between 5 and 15 min). In this protocol, participants evolved through three progressive stages in terms of volume and intensity, until symptom free: (1) 5 min progression every two sessions, from 5 to 15 min of low-intensity aerobic activity (perceived exertion of 2/10), (2) 5 min progression every two sessions, from 15 to 20 minutes of moderate-intensity aerobic activity (perceived exertion of 3/10), and (3) 5 min of low-intensity aerobic activity (perceived exertion of 2/10), followed by three-to-five low- to high-intensity intervals (alternate 1 min at high-intensity (perceived exertion of 5/10) and 1 min at low-intensity aerobic activity (perceived exertion of 2/10)), followed by 5 min low-intensity aerobic activity (perceived exertion of 2/10). The onset or increase of symptoms led to immediate termination of exercise for a minimum of 24 hours. Patients were asked to return to the previous stage if any of the symptoms increased by 1 point on a 10-point symptoms intensity scale during or after exercise. They had to progress through stages if they successfully completed a stage twice in a row. They were instructed not to exercise for two consecutive days.

#### 2.3.2. Sport-Specific Coordination Exercises

Participants had to perform low-intensity (2/10) sport-specific coordination skills (shooting drills, dribbling drills, agility, etc.) for 5 to 10 min after every session of aerobic training. The activity had to be stopped if it generated any symptoms.

#### 2.3.3. Therapeutic Balance Exercises

Participants were asked to perform three 30-seconds repetition of three therapeutic exercises consisting in balance exercises (standing on one foot with eyes closed, feet in tandem on an unstable surface, etc.) (Figure 3).

The home program included all three components of the Active Rehabilitation Intervention program and was provided to the families on paper. Progression was monitored through a weekly follow-up phone call. The participants and parents were asked to contact the study coordinator for any questions or if symptoms worsened or resolved. Follow-up was conducted until the participant reported being symptom-free for at least 7 days.

### 2.4. Participation in the Active Rehabilitation Intervention

Participation in the Active Rehabilitation Intervention was monitored through weekly follow-up phone calls and was determined by comparing a participant's report of exercises sessions performed with exercise sessions prescription (%). Correlation between the duration of the intervention and self-reported participation was calculated to evaluate the dose-response relationship.

### 2.5. Signs and Symptoms Assessment

#### 2.5.1. Symptoms Assessment

Current postconcussion symptoms and preinjury symptoms severity were assessed by the Postconcussive Symptom Inventory scale (PCSI) [42]. The PCSI is a validated questionnaire of 21 items rated from 0 to 6 (0 = absence of symptom and 6 = severe problem) for youth of 13 to 18 years old who sustained a mTBI, where item 21 is a self-reported abnormality score rated from “0” indicating “No Difference” to “4” indicating “Very Different.” The first 20 items' scores were added in order to create the PCSI score (0–120 points). The 18-item PCSI questionnaire form was used for participants under 13 years of age. Parents were asked to complete the PCSI parent version to rate their children's perceived symptom severity. Pre- and postintervention PCSI scores and self-reported abnormality scores were compared for each participant and parent and between the participant and their parent. The PCSI score results for participants #3 and #11 have not been considered in the statistical analysis due to the use of incompatible versions of the questionnaires.

#### 2.5.2. Neuropsychological Assessment

A battery of neuropsychological tests was used to assess multiple aspects of cognitive functioning, including verbal memory (Rey Auditory Verbal Learning Test (RAVLT) [47]), verbal fluency (Delis-Kaplan executive function system (D-KEFS) [48]), working memory (Digit Span from the Weschler Adult Intelligent Scale (WAIS-IV) [49] or from the Weschler Intelligence Scale for Children (WISC-IV) [50]), information processing (Symbol Digit Modality Test (SDMT) [51]), and attention processes (Continuous Performance Test II (CPT-II) [52]). The tests were selected to evaluate the most affected cognitive functions following an mTBI as reported in the literature [53, 54]. This neuropsychological test battery was administered by a trained neuropsychologist. The test administration was standardized and uniform for all participants.

A psychological inventory (State-Trait Anxiety Inventory (STAI) [55]) was also administered to the participants' parents to measure their anxiety about their child's condition and their anxiety level as a personal characteristic [55].

#### 2.5.3. Coordination and Balance

Coordination and balance were assessed by subtests from the Sport Concussion Assessment Tool v3 (SCAT3) Balance examination tests and coordination examination [1] through the Modified Balance Error Scoring System (BESS) testing, the tandem gait and the Finger-to-nose task. Participants performed the Modified Clinical Test of Sensory Interaction on Balance (m-CTSIB) [56], which measures static postural sway, and the Limit of Stability test (LOS), which measures dynamic postural control within a normalized sway envelope on a Biodex Biosway™ force platform (Biodex Medical Systems, Inc. Shirley, NY, USA) [57]. Finally, balance and coordination were also assessed by the Bruininks-Oseretsky Test of Motor Proficiency 2nd Edition (BOT-2) subtests of bilateral coordination, balance, and upper-limb coordination [58].

### 2.6. Statistical Analysis

Spearman correlation between the duration of the intervention and self-reported participation in the Active Rehabilitation Intervention and between Active Rehabilitation Intervention duration and time post mTBI preintervention were calculated. The Wilcoxon non parametric test for related samples was conducted on every other outcome measures. Outcome measures statistical analyses were conducted with the Statistical Package for the Social Sciences (SPSS) version 22 (SPSS. Inc., Chicago, Illinois, USA). BOT-2 subtests total point scores were converted to derived scores in order to have uniform meaning between subtests and participants. Normalized age and sex scale scores were used for statistical analysis.

## 3. Results

All participants were previously engaged in organized recreational to high performance sport groups for 6 ± 4 hours per week. Most sustained a sport-related mTBI in their principal competitive activity (*n* = 14) or in another recreational activity (*n* = 2). Two participants sustained a motor vehicle-related incident. From the previous 18 participants screened, 15 children and adolescents (9 females) aged 15.00 ± 1.69 years were included in this study. Three participants were excluded from the data analysis for the following reasons: one participant was not able to complete the final assessment because he suffered from a fractured toe and therefore decided to quit the rehabilitation program and two other participants returned to their sport without the consent of their treating clinicians, although they were still experiencing postconcussion symptoms. Participant characteristics are presented in Table 1. All participants had a normal academic progress and reported no cognitive, behavioural, neurological, or musculoskeletal impairments. Participant #4 was treated with carbamazepine (Mylan-Carbamazepine CR) for epilepsy. Participant #6 was diagnosed with influenza during the study and had flu symptoms for 10 days, after being asymptomatic for five days. Participant #7 suffered from iron deficiency anaemia and anxiety disorder, had a history of migraines and was treated with iron supplements, almotriptan (Axert®), and amitriptyline (Elavil®). No participants had sustained an mTBI in the previous 6 months before data collection. Seven participants reported amnesia of the event, three had lost consciousness, and five had sought medical attention at the emergency department. Participants #3 and #12 had an impact to the head during the study without convincing signs of mTBI. Screening and treatment for associated conditions of cervical (*n* = 13), oculomotor (*n* = 10), vestibular (*n* = 5), and temporomandibular (*n* = 1) dysfunctions were conducted by a registered physiotherapist.

### 3.1. Intervention Duration before Recovery

Participants were initially evaluated at 48 ± 88 days after mTBI. The intervention lasted 49 ± 17 days before recovery was attained. The final evaluation was conducted after 9 ± 2 days without symptoms (Table 1). Spearman correlation revealed no correlation between the Active Rehabilitation Intervention duration and the duration of postconcussion symptoms before the intervention (*r* = −0.120, *p* = 0.671).

### 3.2. Participation in the Active Rehabilitation Intervention

**Figure 2:**
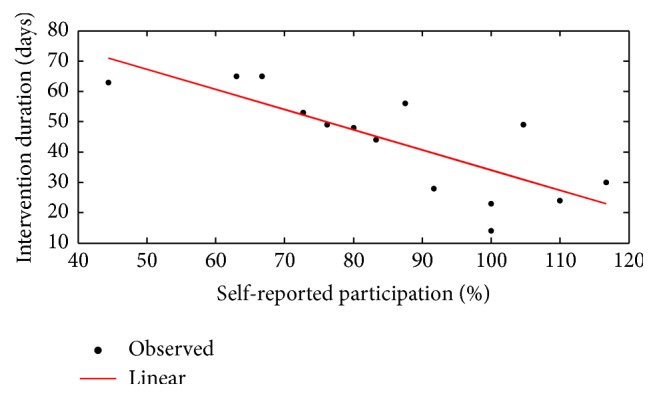
Correlation of duration of the intervention to self-reported participation.

**Figure 3:**
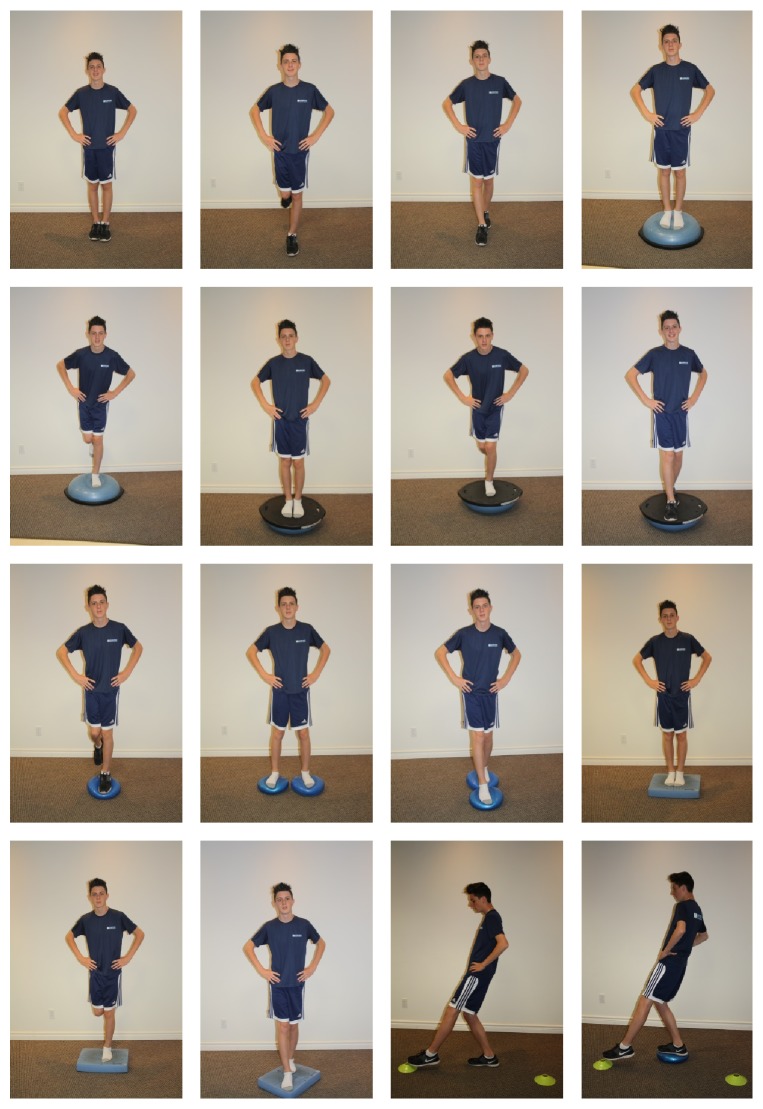
Therapeutic exercises.

Participants completed on average 85 ± 20% of the Active Rehabilitation Intervention sessions prescribed. Duration of the intervention was correlated with self-reported participation score (*r* = −0.796, *p* ≤ 0.001) (Figure 2). Three participants did the Active Rehabilitation Intervention program 3 to 4 times a week (every two days) which explained participation score higher than 100%.

### 3.3. Symptoms

Symptom frequency and severity decreased significantly after the intervention (see Table 2). Before the intervention, the participants reported an average of 8.9 ± 2.1 symptoms on the PCSI. The number of self-reported symptoms decreased significantly to 1.8 ± 1.4 following the intervention (*Z* = −3.933, *p* ≤ 0.001). The most frequently symptoms from the pretest were fatigue (*n* = 13), difficulty concentrating (*n* = 12), headache (*n* = 11), and answering questions more slowly than usual (*n* = 11). The PCSI score went from a total of 36.9 ± 23.2 points before the intervention to 4.3 ± 5.0 points after the intervention (*Z* = −3.18, *p* = 0.001). The self-reported abnormality score (PSCI item 21) decreased significantly from 2.0 ± 3.3 points to 0.2 ± 0.4 points (*Z* = −2.88, *p* = 0.004). Parents reported similar decrease in symptom perception of the PCSI score from 38.8 ± 20.3 to 12.8 ± 9.6 points (*Z* = −2.904, *p* = 0.004). They reported an abnormality score (item 21) of 2.1 ± 0.7 and 0.6 ± 0.5 points before and after the intervention, respectively (*Z* = −2.879, *p* = 0.004). Abnormality scores were similar between participants and their parents (*Z* = −1.342, *p* = 0.180).

### 3.4. Neuropsychological Results

The results of the neuropsychological test assessment are summarized in Table 3. Participants showed better performance in verbal episodic memory, both in total learning (*Z* = −2.843, *p* = 0.004) and in immediate recall (*Z* = −2.68, *p* = 0.001), in switching semantic verbal fluency (*Z* = −2.147, *p* = 0.032) and working memory (operational component (backward)) (*Z* = −2.54, *p* = 0.011) and they made less omission type errors in a task measuring different attention processes (*Z* = −2.386, *p* = 0.017) after the Active Rehabilitation Intervention. Other neuropsychological subtests did not differ when compared to the initial evaluation. No correlations were found between the Active Rehabilitation Intervention duration and the parental anxiety measured by the STAI for state anxiety (*r* = 0.112, *p* = 0.692) and trait anxiety (*r* = 0.042, *p* = 0.881).

### 3.5. Coordination and Balance

Coordination and balance results are presented in Table 4. Modified Balance Error Scoring System (BESS) on one leg (*Z* = −3.068, *p* = 0.002) and tandem (*Z* = −2.149, *p* = 0.032) stances, tandem gait (*Z* = −3.233, *p* = 0.001), and the Finger-to-nose (*Z* = −3.234, *p* = 0.001) scores improved after the intervention. Modified Clinical Test of Sensory Interaction on Balance (m-CTSIB) under the conditions:* Eyes opened, firm surface* (*Z* = −2.217, *p* = 0.027),* Eyes opened, foam surface* (*Z* = −2.309, *p* = 0.021) and* Eyes closed, foam surface* (*Z* = −3.068, *p* = 0.002) were significantly improved. Limit of Stability (LOS) improved from 56.6 ± 13.1% to 70.8 ± 9.9% (*Z* = −3.068, *p* = 0.002). Finally, the BOT-2 subtests of* bilateral coordination *(*Z* = −2.673, *p* = 0.008) and* upper-limb coordination *(*Z* = −3.121, *p* = 0.002) improved significantly after the intervention while the* balance* subtest remained unchanged (*Z* = −0.841, *p* = 0.400).

## 4. Discussion

The present study investigated the effects of an Active Rehabilitation Intervention integrated with standard care [1] on recovery, assessed by a postconcussion symptom inventory, cognitive function, and coordination and balance performance in a slow-to-recover paediatric population following a mTBI. All participants reported full recovery. The duration of the intervention was correlated with self-reported participation. The majority of coordination and balance assessment tests presented a significant improvement following the intervention.

Since postconcussion symptoms are not specific to mTBI, some patients may report symptoms on PCSI even if they report having recovered from their mTBI [42]. In this study, participant's self-reported abnormality score normalized after the Active Rehabilitation Intervention. Although there is a need to develop accessible and valid interventions to improve recovery in paediatric mTBI population, the literature supports the use of exercise as an Active Rehabilitation Intervention when symptoms persist beyond the expected recovery period after mTBI in adults [25, 26]. To date, only three similar studies conducted such interventions in youth [9, 35, 45]. Our intervention was inspired by the Montréal Children's Hospital Trauma Centre Mild Traumatic Brain Injury Program [9, 45]. Although our intervention differs in frequency of participation per week and in intensity/duration of aerobic exercise reached at the end of the program, the total duration of the Active Rehabilitation Intervention before recovery (49 ± 17 days) was similar to those observed by others [9, 45]. The Active Rehabilitation Intervention duration was not correlated with duration of autoreported postconcussion symptoms since the last mTBI (*r* = 0.205, *p* = 0.464). Considering that all participants reported a complete recovery from mTBI after the intervention, it is possible that physical activity had promoted recovery after mTBI, although recovery time overlaps this, as observed by Barlow et al. [8]. Moreover, there is an apparent dose-response relationship between duration of the intervention and self-reported participation (*r* = −0.792, *p* ≤ 0.001). This leaves us to believe in the effectiveness of exercise to decrease postconcussion symptoms. On the other hand, we cannot rule out the possibility of a bias created by the self-reported nature of the participation assessment.

Neuropsychological results suggest that athletes showed better performance in some verbal episodic memory, verbal fluency, working memory (operational component), and attention processes after the Active Rehabilitation Intervention. These results are consistent with the literature, indicating that episodic verbal memory, verbal fluency, working memory, and attention processes appear to be affected following a mTBI [53, 54]. Although there is a significant improvement in some parameters of cognitive functions, other parameters were not affected by the intervention (i.e., verbal memory (ListB, Delayed), verbal fluency (phoenemic, semantic, semantic switching), working memory (forward), information processing, and attention processing (commission, hit, Hit reaction time, variability, and detectability)). This finding may be explained by the fact that athletes initially did not show any cognitive impairment. It may also relate to an incomplete recovery process, although participants report a subjective improvement in terms of number and intensity of autoreported symptoms. This is consistent with the literature indicating that although, in most cases, cognitive recovery largely overlaps with the time course of symptom recovery, it may occasionally follow clinical symptom resolution [1, 59, 60]. However, the limitations of the study, including the use of a small sample size and the use of alternative forms of neuropsychological tests, which could have induced a learning effect on the posttest measures, may have reduced the scope of this study. Participants demonstrated a significant improvement in coordination and balance following the intervention. These results are consistent with the literature indicating that coordination and balance are known to be altered after a mTBI in youth and young adults [53, 61–64]. The coordination and balance components of the Active Rehabilitation Intervention could impact the coordination and balance assessment results under the effect of practice. Therefore, the interpretation of the results must be made cautiously.

The lack of a control group and the limited number of participants do not allow us to draw conclusions about the effectiveness of this intervention. However, it is likely that maintaining physical rest is not optimal when the recovery process is longer than expected in youth. No participant reported having a deterioration of their condition following the Active Rehabilitation Intervention. Therefore, it is likely that the initiation of progressive low- to high-intensity physical activity, without risk of fall or impact, is safe in young athletes one month following mTBI. Physiotherapy treatments may also have had an impact on physical postconcussive symptoms.

## 5. Conclusion

A progressive submaximal Active Rehabilitation Intervention may be an important asset in the recovery in young patients who are slow-to-recover following a mTBI. The majority of coordination and balance assessment tests and some cognitive functions, such as working memory, verbal memory, verbal fluency, and attention processes, presented a significant improvement following the Active Rehabilitation Intervention. Symptoms resolved in every participant independently of the onset of the Active Rehabilitation Intervention in the recovery process. It is unclear how the Active Rehabilitation Intervention has influenced these results. This study is a step toward the integration of a multimodal rehabilitation intervention in patients who are slow-to-recover after a mTBI. Further studies including more participants and a control group are needed to validate this promising new approach. This study has the potential to influence traumatology practice and may promote sports therapy in mTBI rehabilitation, although additional research in this area is needed.

## Figures and Tables

**Table 1:** Participants characteristics.

Participants	Age (years)	Sex	Weight (kg)	Height (m)	Number of previous mTBIs	Amnesia	LOC	Sport	Duration of postconcussion symptoms before the intervention (days)	Active rehabilitation duration (days)	Time without symptoms after intervention (days)
01	15.0	F	46.8	1.60	1	Yes	No	Soccer	60	53	9
02	16.0	F	50.9	1.63	1	Yes	No	Basketball	86	44	10
03	12.3	M	38.6	1.56	2	No	No	Hockey^*∗*^	30	27	13
04	13.6	F	47.7	1.58	0	Yes	Yes	Gymnastics	30	30	7
05	14.8	M	72.7	1.78	2	No	No	Hockey	48	49	9
06	15.0	M	75.9	1.73	1	No	No	Basketball	41	65	7
07	17.5	F	65.0	1.67	1	Yes	No	Synchronized swimming^*∗*^	158	53	8
08	14.0	F	61.4	1.72	0	No	No	Soccer	373	63	9
09	15.8	M	92.3	1.83	2	Yes	Yes	Football^*∗∗*^	120	14	10
10	15.3	F	66.4	1.71	0	Unclear	No	Basketball	60	24	14
11	11.3	M	36.4	1.39	2	No	No	Hockey	31	65	8
12	15.4	F	53.6	1.58	1	No	No	Soccer	30	56	9
13	17.1	M	70.5	1.66	0	Yes	Yes	Snowboarding	64	23	13
14	15.0	F	54.5	1.64	0	No	No	Soccer	36	48	10
15	16.8	F	81.8	1.67	0	Unclear	No	Cycling^*∗∗*^	45	49	10

^*∗*^Motor vehicle-related mTBI.

^*∗∗*^Sport-related mTBI in another recreational sport.

LOC: loss of consciousness.

**Table 2:** Participants' postconcussion symptom inventory results.

	Before intervention	After intervention	*Z*	*p*
PCSI score (sum of 20 items)^*∗*^	36.9 ± 23.2	4.3 ± 5.0	−3.180^b^	0.001
Self-reported abnormality score^*∗*^	2.0 ± 3.3	0.2 ± 0.4	−2.877^b^	0.004

^*∗*^Significant statistical difference.

^b^Based on positive ranks.

**Table 3:** Neuropsychological results.

	Before intervention	After intervention	*Z*	*p*
RAVLT				
Trials 1 to 5 total^*∗*^	47.46 ± 6.18	54.38 ± 7.34	−2.843^b^	0.004
List B interference	4.76 ±1.59	6.23 ± 1.36	−1.736^b^	0.083
Immediate recall^*∗*^	9.77 ± 2.20	11.54 ± 1.90	−2.568^b^	0.001
Delayed recall	10.08 ± 2.66	11.15 ± 2.27	−1.724^b^	0.085
Verbal fluency (words)				
Phonemic	26.60 ± 7.23	29.60 ± 9.43	−1.449^b^	0.147
Semantic	37.64 ± 4.24	35.21 ± 4.81	−1.749^c^	0.080
Semantic switching (total responses)^*∗*^	14.50 ± 2.21	11.93 ± 2.62	−2.147^c^	0.032
Semantic switching (total switching)	13.64 ± 2.37	11.36 ± 2.41	−1.891^c^	0.059
Digit span				
Forward	9.14 ± 1.70	9.29 ± 1.49	−0.426^b^	0.670
Backward^*∗*^	7.43 ± 1.10	8.64 ± 1.91	−2.537^b^	0.011
SDMT				
Total	53.43 ± 7.59	56.86 ± 9.53	−1.857^b^	0.063
CPT II				
Omission^*∗*^	4.33 ± 3.94	1.18 ± 1.17	−2.386^c^	0.017
Commission	23.92 ± 10.08	19.81 ± 7.08	−1.532^c^	0.126
Hit	380.80 ± 56.99	369.48 ± 35.67	−0.533^c^	0.594
Hit reaction time	6.10 ± 2.66	4.73 ± 1.24	−1.600^c^	0.110
Variability	10.04 ± 7.23	6.32 ± 1.61	−0.800^c^	0.424
Detectability	0.42 ± 0.585	0.52 ± 0.33	−1.512^c^	0.130

^*∗*^Significant statistical difference.

Note: RAVLT, Rey auditory verbal learning test; SDMT, symbol-digit modalities test; CPT, Continuous Performance Task.

^b^Based on negative ranks.

^c^Based on positive ranks.

**Table 4:** Coordination and balance.

	Before intervention	After intervention	*Z*	*p*
SCAT3				
BESS double leg stance	0.07 ± 0.26	0.00 ± 0.00	−1.000^b^	0.317
BESS one leg stance^*∗*^	5.20 ± 2.96	1.73 ± 2.63	−3.068^b^	0.002
BESS tandem stance^*∗*^	2.80 ± 2.48	1.20 ± 2.01	−2.149^b^	0.032
Tandem gait^*∗*^	17.93 ± 7.89	13.21 ± 4.08	−3.233^b^	0.001
Finger-to-nose^*∗*^	3.54 ± 0.55	3.00 ± 0.48	−3.234^b^	0.001
Biosway				
mCTSIB eyes opened firm surface^*∗*^	0.53 ± 0.27	0.39 ± 0.17	−2.217^b^	0.027
mCTSIB eyes closed firm surface	1.12 ± 0.88	0.69 ± 0.21	−1.874^b^	0.061
mCTSIB eyes opened foam surface^*∗*^	0.78 ± 0.21	0.67 ± 0.19	−2.309^b^	0.021
mCTSIB eyes closed foam surface^*∗*^	2.27 ± 0.60	1.87 ± 0.46	−1.960^b^	0.050
LOS^*∗*^	56.6 ± 13.14	70.8 ± 9.92	−3.068^c^	0.002
BOT2				
Bilateral coordination^*∗*^	12.33 ± 4.69	16.07 ± 4.03	−2.673^c^	0.008
Balance	12.07 ± 4.82	13.27 ± 4.43	−0.841^c^	0.400
Upper-limb coordination^*∗*^	13.73 ± 4.08	18.33 ± 4.03	−3.121^c^	0.002

^*∗*^Significant statistical difference.

^b^Based on positive ranks.

^c^Based on negative ranks.
